# Cortico-subcortical networks that determine behavioral memory renewal are redefined by noradrenergic neuromodulation

**DOI:** 10.1038/s41598-025-93263-3

**Published:** 2025-03-20

**Authors:** Josue Haubrich, Laura Dolón Vera, Denise Manahan-Vaughan

**Affiliations:** Medical Faculty, Department of Neurophysiology, Ruhr University Bochum, Universitätsstr. 150, MA 4/150, 44780 Bochum, Germany

**Keywords:** Spatial memory, Extinction, Neural circuits, Neuroscience, Neurotransmitters

## Abstract

During spatial appetitive extinction learning (EL), rodents learn that previously rewarded behavior is no longer rewarded. Renewal of the extinguished behavior is enabled by re-exposure to the context in which rewarded learning occurred. When the renewal response (RR) is unrewarded, it is rapidly followed by response extinction (RE). Although the hippocampus is known to be engaged, whether this dynamic is supported by different brain networks is unclear. To clarify this, male rats engaged in context-dependent spatial memory acquisition, EL and RR testing in a T-Maze. Fluorescence in situ hybridization disambiguated somatic immediate early gene expression in neuronal somata engaged in RR or RE. Graph analysis revealed pronounced hippocampal connectivity with retrosplenial and prefrontal cortex (PFC) during initial RR. By contrast, RE was accompanied by a shift towards elevated coordinated activity within all hippocampal subfields. Given that β-adrenergic receptors (β-AR) regulate spatial memory, we activated β-AR to further scrutinize these network effects. This *enhanced* RR and prevented RE. Effects were associated with initially increased thalamic-hippocampus activity, followed by a decrease in hippocampal intraconnectivity and the predominance of network activity within PFC. Our findings highlight a critical hippocampal-cortical-thalamic network that underpins renewal behavior, with noradrenergic neuromodulation playing a pivotal role in governing this circuit’s dynamics.

## Introduction

Effective adaptation to a dynamically changing world necessitates the integration of new, or updated, information into pre-existing memories of past experience. Recognising that a previous association, or cue, no longer leads to an expected outcome requires new learning that is referred to as extinction learning (EL)^1,2^. EL can occur in the absence of a change in context, but is more effective when a context-change signals that circumstances have changed^3,4^. This competition between old and new knowledge, that drives the encoding of apposite, but opposing, information, can be influenced by both external contextual cues^3^, as well as neuromodulation^5,6^.

EL processes do not only address the revision of fearful or aversive behavior, but also encompass appetitive and positive experiences^7,8^. This aspect of the EL research field is under-represented compared to research into aversive EL, but reflects a highly relevant feature of behavior that predominates in daily life. Most forms of benign associative experience, including appetitive spatial learning, require active updating, leading to behavioral consolidation or adaptation, during retrieval^9,10^. EL and renewal of benign associative experience is an essential facet of prospective thinking and future decisions^11^. Thus, in contrast with approaches that aim to suppress and understand the mechanisms underlying renewal of aversive experience from a therapeutic perspective^12^, renewal of a previously learned benign experience is a highly relevant and necessary mechanism when EL is only of temporary significance. For example, one learns that a once-familiar route to a bakery is no longer worthwhile because the bakery has temporarily closed (EL). However, when the bakery reopens, the original route becomes advantageous again, prompting the reactivation of the memory of the previously used route and a return to this strategy (renewal). The mechanisms underlying these highly pertinent processes deserve close scrutiny. Cellular processes, such as hippocampal synaptic plasticity, support this kind of information updating and adaptation^13^, but very little is known about network activity that underlies EL and renewal of appetitive spatial learning.

The hippocampus supports EL and renewal of spatial appetitive experience^14^, possibly through the updating of episodic memory^15^. This is largely unsurprising, given that it is a brain region that plays a crucial role in the encoding, retrieval, and updating of memories of both positive and negative valence^16–18^. Additionally, the hippocampus plays a pivotal role in spatial-contextual information processing^19,20^ and its DG-CA3-CA1 circuitry computes both pattern separation and completion^21,22^, as well as discrete elements of spatial and non-spatial content^23^, thereby supporting the readjustment of cue-outcome relationships resulting from contextual changes.

It seems unlikely that the hippocampus operates alone during EL and renewal of spatial appetitive experience. It is embedded in a wider network of interconnected brain structures that enable flexible memory formation and retrieval^17^. Complementing the hippocampus’ function in memory and navigation, the retrosplenial cortex (RSC) dynamically processes spatial, cognitive and reinforcement information^24^. The ventral hippocampus interfaces both anatomically and functionally with the prefrontal cortex (PFC)^25^. The infralimbic (IL) region of the PFC is involved in decision-making, response adaptation, and in facilitating EL^26,27^, whereas the prelimbic (PL) region counteracts these processes^28,29^. In addition, thalamic nuclei relay and process sensory information^30^, support memory functions^31,32^, as well as promote directed attention^33^, and therefore comprise integral components of memory-related networks. Specifically, the paraventricular nucleus (PV) of the thalamus integrates prior experiences with current homeostatic states to modulate motivated behaviors^34^, while the ventrolateral and dorsomedial portions of the laterodorsal nucleus (LDVL and LDDM, respectively) receive extensive visual inputs and contribute to spatial navigation and the consolidation of memory^35,36^. For this reason, network activity in the abovementioned structures during renewal of spatial appetitive experience was the focus of this study.

It has been proposed that consolidated associative memory is stored in distributed cortico-cortical structures^37^. Nonetheless, the hippocampus becomes engaged during retrieval of previously consolidated experiences^38^, including memory retrieval following spatial appetitive EL^14^. To what extent the hippocampus becomes activated during renewal of spatial appetitive experience, and which cortical and subcortical structures are involved in this process is largely unknown. A key motivation behind the current study was, therefore, the question as to how renewal of spatial appetitive experience is represented on a functional network level. To do this, we used fluorescence *in situ* hybridisation (FISH) to detect somatic expression of immediate early genes (IEG)^23^ that was triggered during the early and late phases of unrewarded renewal of spatial appetitive experience. We applied graph theory analysis to detect hubs of connectivity during these processes^39,40^. By this means we could pinpoint cortical areas, thalamic nuclei, and hippocampal subfields that were directly involved in the initial renewal response and in the late extinction of the renewed behavior, that occurred because a reward was not present.

It is known that noradrenaline (NA) acting on β-adrenergic receptors (β-AR) potently regulates hippocampal information processing, and storage^41–43^ and is particularly involved in learning and memory^42,44^. For instance, research using various paradigms has demonstrated that β-AR activation is involved in memory consolidation^45–47^, and reconsolidation^48,49^. Because of its widespread influence and potent effect on local circuits, β-AR activation can also dictate patterns of brain function at the network level^43^. To what extent β-AR activation affects the state of brain-wide networks during memory retrieval in situations where contextual information brings ambiguity, such as in memory renewal of an unrewarded experience following EL, is unclear.

For this reason, we examined how intracerebral treatment of the animals with a β-AR agonist, prior to renewal testing, affected network dynamics. The question was whether β-AR activation would promote renewal or the extinction of the renewal response. This question was motivated by the controversy in treatment outcomes of post-traumatic stress disorder with β-AR ligands^50^ and our own findings that pharmacological antagonism of β-AR can reinforce renewal of spatial appetitive experience^51^. Furthermore, NA release in the hippocampus promotes network reset^52^ as well as memory-updating^42^ and episodic-like memory in rodents^47^.

β-AR activation by means of its agonist isoproterenol has been shown to promote hippocampal synaptic plasticity^46,54–56^ and memory consolidation^57^ over extended periods, even though this agonist has a short half-life^53^. These effects are hypothesized to involve downstream cascades that generate glutamate-NA hotspots, prioritizing the processing of highly relevant information^58^. For example, isoproterenol treatment increases CREB phosphorylation for up to 30 min and cfos mRNA expression for at least 60 min^59^, and also increases AMPA receptor phosphorylation and recruitment for at least one hour^60^.

Thus, the goal of our study was to discriminate network activity in brain areas that contribute to initial renewal of a spatial appetitive experience, followed by EL of this response when animals realise that no reward is available. In addition, we examined the involvement of β-AR in this process by using its agonist isoproterenol, as a model of state-dependent modulation of retrieval and updating dynamics of spatial appetitive experience. We identified a critical hippocampal-cortical-thalamic network that orchestrates the renewal of extinguished behavior, in the absence of a reward,  and found that response extinction after renewal is associated with predominant network activation within the hippocampus. Furthermore, β-adrenergic signaling promotes the rerouting of network activity to sustain the renewal of extinguished behavior. Our findings suggest that state-dependent differences in the renewal response are potently mediated by β-adrenergic signaling, whereby NA acting on these receptors supports persistence of renewal by driving a decline in coordinated connectivity and promoting a state of parallel, rather than integrated processing across brain regions.

## Results

### β-AR activation prolongs context-dependent renewal of extinguished responses and prevents response extinction.

**Fig. 1:**
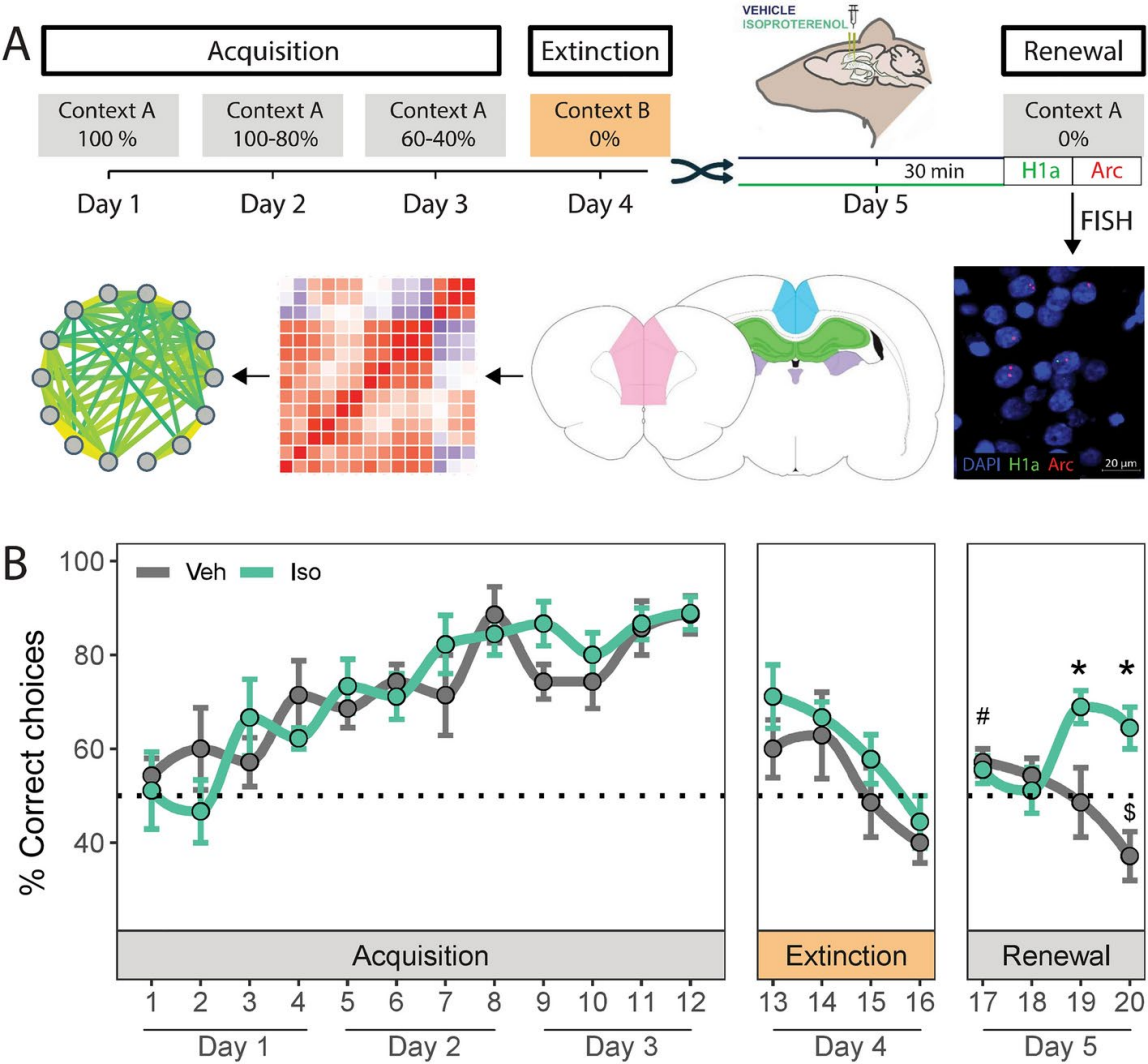
Context-dependent spatial memory renewal is boosted by β-AR activation. **A**) Experimental design: Rats were trained to locate a reward in a T-Maze in context A and underwent an EL session in context B. Next, animals were assigned to receive icv infusions of either vehicle solution (Veh) or the β-AR agonist, isoproterenol (Iso), and then underwent renewal testing in context A. Neuronal activation throughout renewal testing was assessed by quantifying H1a + and Arc + nuclei using fluorescence *in situ* hybridization. The interregional correlations between all pairs of structures were computed followed by graph analysis of functional network connectivity. **B**) Behavioral performance in the T-Maze: Animals of both groups equally learned to pursue the rewarded location during the acquisition session and extinguished this behavior in the extinction session. During renewal testing, the recovery of the extinguished responses was higher in the Iso group. Plot shows mean ± s.e.m. N = 9/10 per group. Asterisks (*) indicate statistical significance between groups within the same testing block, # indicates significant within-group differences between block 16 and 17 in both groups, and $ indicate significant within-group differences between block 17 and 20 in the vehicle group, at p < 0.05.

We investigated brain networks supporting the recovery of extinguished spatial appetitive responses by examining somatic expression of Homer1a to detect neurons that engaged in response renewal and somatic expression of Arc to detect neurons that engaged in response extinction (Fig. 1A). To examine the role of state-dependent neuromodulation, renewal was compared in animals treated intracerebrally (icv) with a β-AR agonist or vehicle. Rats underwent a 3-day acquisition phase to learn to locate a low probability reward in a T-Maze using spatial cues (context “A”), followed by an EL session on day 4 in context “B” where the reward was removed, and contextual features of the maze were changed (e.g. ambient lighting tone—warm or cold, without altering light intensity, altered floor pattern, contrast and features of distal cues, as well as exchanged odor cues). On the next day, the animals were randomized into two groups, with one group receiving bilateral icv infusions of vehicle solution (Veh) and the other group receiving the β-AR agonist isoproterenol (Iso). After a 30-min interval, the animals were returned to the T-maze for a renewal testing, where the reward was unavailable, but the context had the same features as during acquisition in context “A”. Here, the reward was absent to create a prediction error that forced the renewal response to be updated, to take into account that the reward contingency was no longer present. We previously showed that this leads to initial response renewal, followed by extinction of this response that is associated with altered hippocampal information processing^14^.

During the acquisition phase in context “A”, the animals gradually developed a preference for the rewarded arm, as evidenced by an increase in the number of correct choices over the training blocks (Repeated measures ANOVA: F_11,187_ = 13.5, p < 0.0001; Fig. 1B). No significant differences occurred between groups (ANOVA: F_1,17_ = 0.69, p = 0.42). During EL in context “B”, both groups behaved equivalently (F_1,17_ = 2.7, p = 0.12), showing a time- and trial-dependent decrease in their correct responses (F_3,51_ = 9.0, p < 0.0001).

During renewal testing in the unrewarded context “A”, an initial significant and similar increase in performance was apparent in both groups (block 16 vs block 17; Repeated measures ANOVA—group: F_1,17_ = 0.06, p = 0.8; block: F_1,17_ = 15.3, p = 0.001), that was consistent with the anticipation of, and search for, a reward in the goal arm. However, during renewal testing as a whole, a significant difference between the two groups was evident (F_1,17_ = 11.8, p = 0.003), as was a group x block interaction (F_3,51_ = 6.4, p = 0.0006).

Tukey post hoc analysis revealed that animals treated with the β-AR agonist displayed a higher number of correct choices in the last two blocks of testing in context “A”, than those treated with vehicle (block 19: p = 0.005; block 20: p = 0.0008). By contrast, after their initial renewal response, animals treated with vehicle displayed response extinction, as evidenced by a significant decrease in correct responses from the first to the last trial (p = 0.01). By contrast, animals treated with the β-AR agonist exhibited enhanced performance in the late phase of testing in context “A” (p = 0.02), consistent with the persistence of the renewal response.

Moreover, the decrease in correct responses during EL was not solely due to incorrect arm entries but also to an absence of arm entries in some animals (“no choices”), indicating task disengagement (Supplementary Fig. 1.A). Compared to late EL, the proportion of “no choices” decreased in both groups during the renewal session, coinciding with the peak renewal effect: in the vehicle group during block 17 (Pairwise Wilcoxon Rank Sum test, p = 0.035), and in the isoproterenol group during block 20 (p = 0.035). To further assess individual performance changes across unrewarded blocks, we normalized correct responses to each animal’s performance during the first EL block (set at 100%). Repeated-measures ANOVA revealed a significant group x block interaction (F_3,42_ = 4.3, p = 0.01). Both groups showed a decrease in normalized correct choices from block 14 (early EL) to block 16 (late EL) (paired t-test, p < 0.05). Vehicle-treated animals recovered their preference during early renewal testing (block 16 vs. block 17: p < 0.05) and re-extinguished it during late renewal testing (block 17 vs. block 20: p < 0.05). In contrast, isoproterenol-treated animals showed a partial response recovery that was not significant during early renewal testing (block 16 vs. block 17: p = 0.08) and exhibited full recovery during late renewal testing (block 16 vs. block 20: p < 0.05) (Supplementary Fig. 1B).

Together, these findings indicate that β-AR activation promotes the renewal of appetitive spatial memory and prevents response extinction in the unrewarded context “A”.

### Spatial memory renewal increases brain activation in hippocampal, cortical and subcortical regions.

**Fig. 2:**
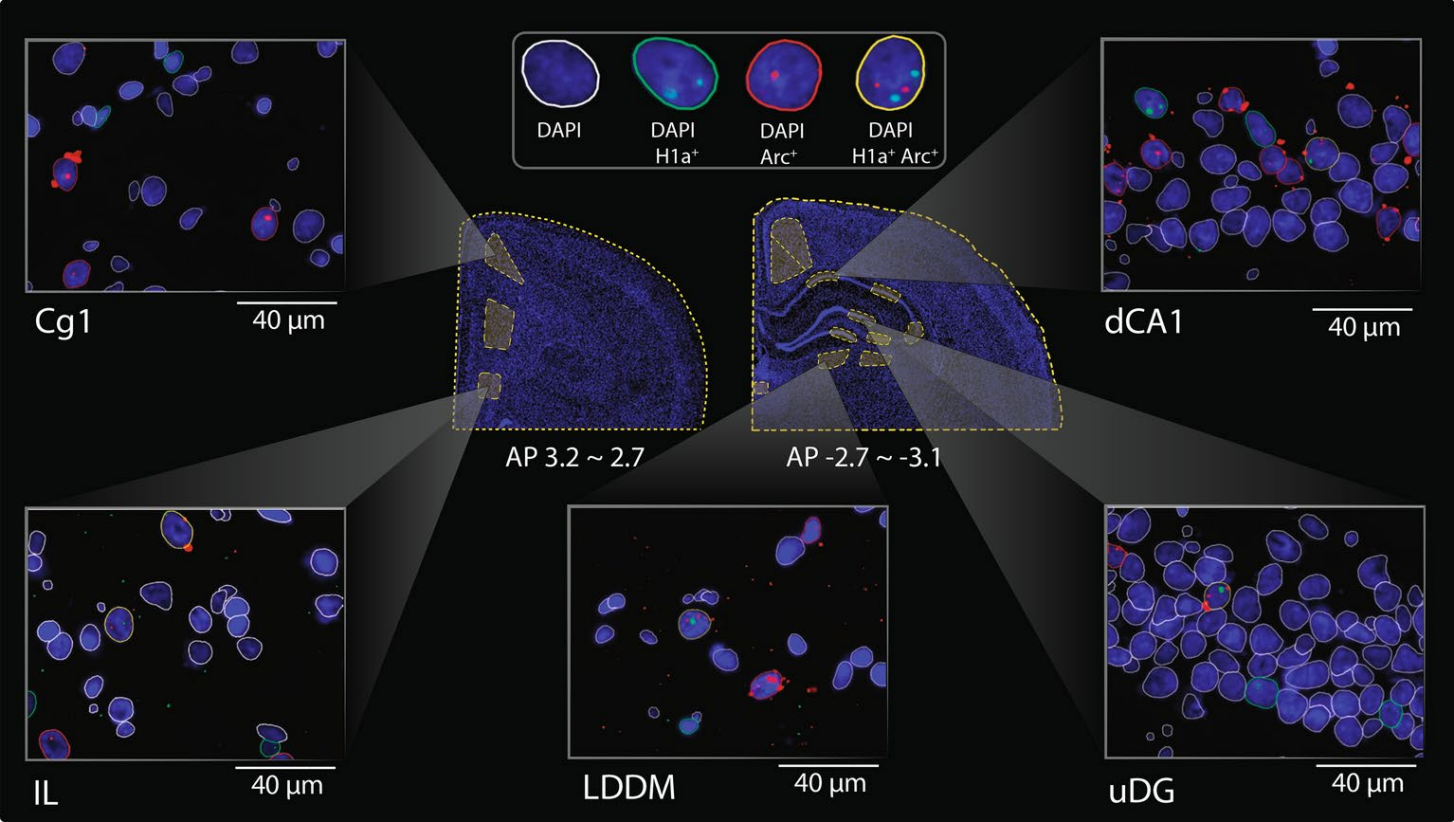
Imaging neural activity during early and late renewal testing using FISH. Fluorescence *in situ* hybridisation (FISH) and time-locked quantification of somatic IEG expression. The brain slice outlines indicate the boundaries of 14 regions selected for IEG quantification (center). Representative images show DAPI-stained nuclei with Homer1a (H1a, green) and Arc (red) foci in five brain areas (square boxes). The top central box illustrates nuclei classification based on IEG detection: DAPI-only (not positive for any IEG marker) and DAPI positive for H1a + , Arc + , and both H1a + and Arc + .

The quantification of immediate early genes (IEG), such as Homer1a, and Arc, in a manner that is time-locked to their peak somatic expression, is an approach that allows the study of neuronal participation in active cognitive experiences, as well as the functional networks of which they are part^61,62^. To reveal neuronal information encoding driven by renewal in context “A”, as well as by response extinction that occurs when the animals realise that no reward is present in this previously rewarded context, we performed fluorescence in situ hybridization (FISH) to quantify neuronal somata that labeled positively for Homer1a and Arc in 14 brain areas (Fig. 2).

By exploiting the unique time course of nuclear RNA expression for different IEGs, FISH can be used to map neuronal activity reflecting experiences occurring at different time points within the same animal^14,23^. Here, somatic Homer1a expression was used as a biomarker for somatic information encoding during initial renewal, and somatic Arc expression was used to capture somatic information encoding during extinction of the renewal response. Home cage controls were also included to provide a reference for the basal expression of these IEGs. The interregional correlations between all pairs of structures were computed and graph network analysis of functional connectivity was conducted.

We examined the following regions: the cingulate cortex (Cg1), prelimbic cortex (PL), infralimbic cortex (IL), the granular and dysgranular layers of the retrosplenial cortex (gRSC and dRSC), the paraventricular nucleus of the thalamus (PV), the ventrolateral and dorsomedial nuclei of the laterodorsal thalamus (LDVL and LDDM), the lower (infrapyramidal) and upper (suprapyramidal) blades of the dentate gyrus (lowDG and upDG, respectively), and the proximal and distal portions of the hippocampal CA3 (pCA3 and dCA3) and CA1 (pCA1 and dCA1) regions (Fig. 2).

**Fig. 3:**
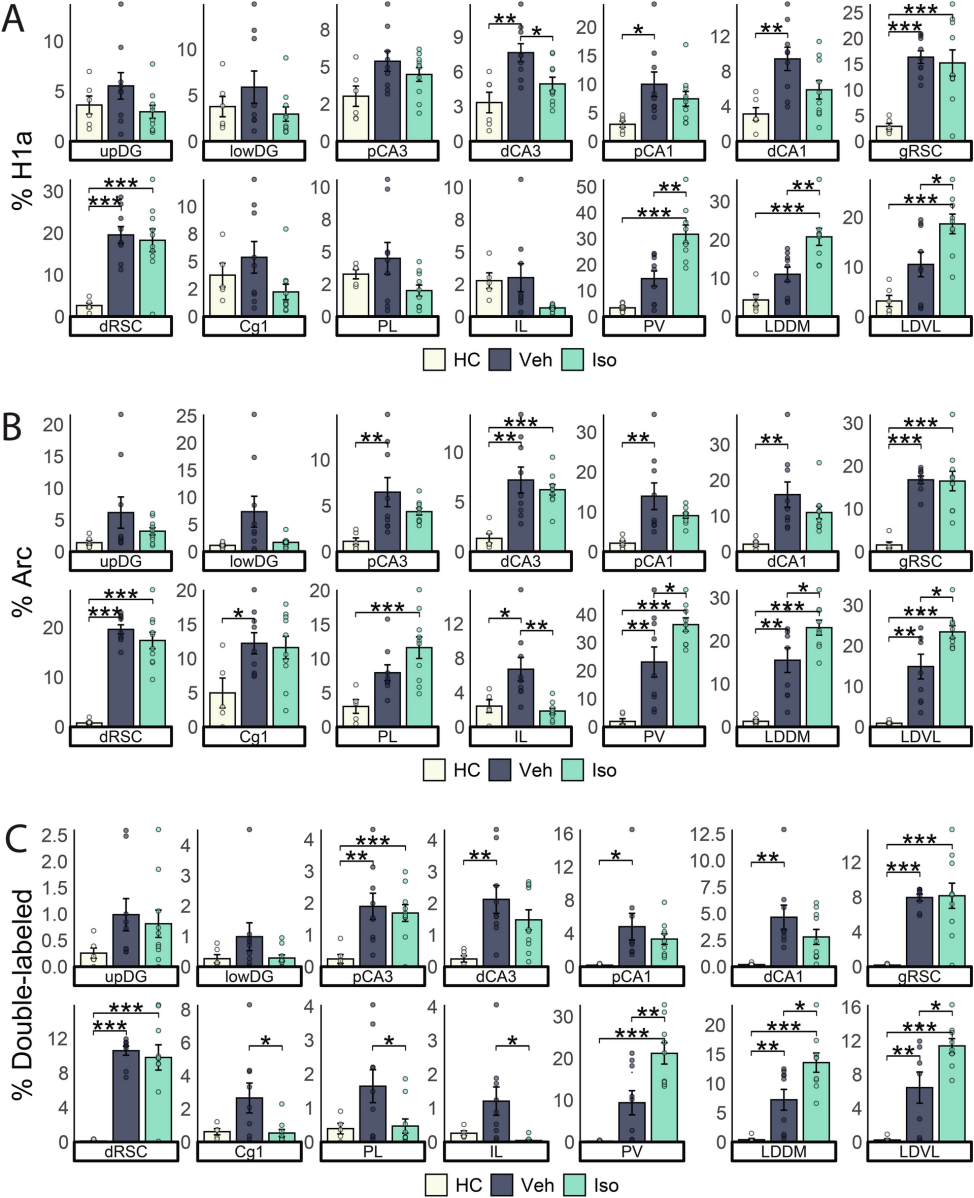
H1a and Arc expression increase across brain regions during renewal and are modulated by β-AR activation. Overall IEG expression across brain areas and experimental groups. **A**) Percentage of Homer1a (H1a)-positive nuclei. **B**) Percentage of Arc-positive nuclei. **C**) Percentage of double-positive nuclei. Plots show mean ± s.e.m. N = 9/10 per group. Asterisks indicate statistical significance at *p < 0.05, **p<0.01, ***p<0.001.

During the early phase of renewal testing in context “A” of control animals, a one-way ANOVA with Tukey’s post-hoc analysis (Fig. 3A, Table 1) indicated that compared to home cage animals, there was an increased Homer1a expression in the dCA3, pCA1, dCA1, gRSC, and dRSC. This suggests an increased activation of brain regions involved in the encoding and retrieval of spatial information. In the late phase of renewal testing, where renewal extinction was evident, Arc expression analysis (Fig. 3B, Table 1) indicated elevated activation in all regions, except for the upDG, lowDG, and PL, revealing increased involvement in information processing of cortical and subcortical regions along with the RSC and hippocampus.

**Table 1:** Comparisons of somatic IEG expression between vehicle-treated and isoproterenol-treated groups with home cage controls.

Group	IEG	upDG	lowDG	pCA3	dCA3	pCA1	dCA1	gRSC	dRSC	Cg1	PL	IL	PV	LDDM	LDVL
Veh	H1a	=	=	=	↑↑	↑	↑↑	↑↑↑	↑↑↑	=	=	=	=	=	=
Veh	Arc	=	=	↑↑	↑↑	↑↑	↑↑	↑↑↑	↑↑↑	↑	=	↑	↑↑	↑↑↑	↑↑
Veh	Double	=	=	↑↑	↑↑	↑	↑↑	↑↑↑	↑↑↑	=	=	=	=	↑	↑
Iso	H1a	=	=	=	=	=	=	↑↑↑	↑↑↑	=	=	=	↑↑↑	↑↑↑	↑↑↑
Iso	Arc	=	=	=	↑↑	=	=	↑↑↑	↑↑↑	=	↑↑	=	↑↑↑	↑↑↑	↑↑↑
Iso	Double	=	=	↑	=	=	=	↑↑↑	↑↑↑	=	=	=	↑↑↑	↑↑↑	↑↑↑

The table shows all pairwise statistical comparisons between vehicle-treated and isoproterenol-treated groups with home cage controls corresponding to the data displayed in Fig. 3. Symbols represent significance as determined by Tukey’s post-hoc tests following one-way ANOVA: ‘ = ’ denotes no significant difference (p ≥ 0.05); ‘↑’ and ‘↓’ indicate significant increases or decreases, respectively, with one arrow indicating p < 0.05, two arrows signifying p < 0.01, and three arrows indicating p < 0.001.

**Cg1**: cingulate cortex; **DG**: dentate gyrus; **dCA1**: distal CA1; **dCA3**: distal CA3; **double**: somata that show labeling of both Arc and Homer1a; **H1a**: Homer1a; **dRSC**: dysgranular retrosplenial cortex; **gRSC**: granular retrosplenial cortex; **IEG**: immediate early gene; **IL**: infralimbic cortex; **Iso**: Isoproterenol; **LDDM**: dorsomedial nuclei of the laterodorsal thalamus; **LDVL**: ventrolateral nuclei of the laterodorsal thalamus; **lowDG**: lower/infrapyramidal blade of DG; **PL**: prelimbic cortex; **pCA1**: proximal CA1; **pCA3**: proximal CA3; **upDG**: upper/suprapyramidal blade of DG; **PV**: paraventricular nucleus of the thalamus; **Veh**: vehicle.

Analysis of the co-expression of Homer1a and Arc was then conducted to quantify cells that were active during both phases of renewal testing (Fig. 3.C). Compared to home cage controls (Table 1), the vehicle group showed more double-labeled cells in the pCA3, dCA3, pCA1, dCA1, gRSC, dRSC, LDDM, and LDVL. This suggests that these structures may engage in information updating during response extinction.

## β-AR activation during renewal changes patterns of brain activation

Next, we evaluated the effect of β-AR activation upon brain activation during renewal. With regard to the early phase of renewal testing, we found higher somatic Homer1a expression in the gRSC, dRSC, PV, LDDM, and LDVL, compared to home cage controls. Relative to vehicle-treated controls, we detected decreased Homer1a expression in the dCA3 and increased expression in the PV, LDDM, and LDVL (Fig. 3A, Tables 1–2). In the late phase of renewal testing, whereby β-AR activation prevented response renewal, we detected elevated nuclear Arc expression in the dCA3, gRSC, dRSC, PL, PV, LDDM, and LDVL, compared to home cage controls. When compared to responses detected in vehicle-treated animals, β-AR activation resulted in decreased nuclear Arc expression in the IL and increased expression in the PV, LDDM, and LDVL (Fig. 3B, Tables 1–2).

**Table 2:** Comparisons of somatic IEG expression between vehicle-treated and isoproterenol-treated groups.

IEG	upDG	lowDG	pCA3	dCA3	pCA1	dCA1	gRSC	dRSC	Cg1	PL	IL	PV	LDDM	LDVL
H1a	=	=	=	↓	=	=	=	=	=	=	=	↑↑	↑↑	↑
Arc	=	=	=	=	=	=	=	=	=	=	↓↓	↑	↑	↑
Double	=	=	=	=	=	=	=	=	↓	↓	↓	↑↑	↑	↑

The table shows all pairwise statistical comparisons between vehicle-treated and isoproterenol-treated groups corresponding to the data displayed in Fig. 3. Symbols represent significance as determined by Tukey’s post-hoc tests following one-way ANOVA: ‘ = ’ denotes no significant difference (p ≥ 0.05); ‘↑’ and ‘↓’ indicate significant increases or decreases, respectively, with one arrow indicating p < 0.05, two arrows signifying p < 0.01, and three arrows indicating p < 0.001. **Cg1**: cingulate cortex; **DG**: dentate gyrus; **dCA1**: distal CA1; **dCA3**: distal CA3; **double**: somata that show labeling of both Arc and Homer1a; **H1a**: Homer1a; **dRSC**: dysgranular retrosplenial cortex; **gRSC**: granular retrosplenial cortex; **IEG**: immediate early gene; **IL**: infralimbic cortex; **Iso**: Isoproterenol; **LDDM**: dorsomedial nuclei of the laterodorsal thalamus; **LDVL**: ventrolateral nuclei of the laterodorsal thalamus; **lowDG**: lower/infrapyramidal blade of DG; **PL**: prelimbic cortex; **pCA1**: proximal CA1; **pCA3**: proximal CA3; **upDG**: upper/suprapyramidal blade of DG; **PV**: paraventricular nucleus of the thalamus; **Veh**: vehicle.

Isoproterenol treatment also resulted in increased numbers of double-labeled neuronal cell bodies in the pCA3, gRSC, dRSC, PV, LDDM, and LDVL compared to home cage controls. In comparison to vehicle-treated animals, isoproterenol treatment reduced double-labeled cells in the Cg1, PL, and IL, but increased them in the PV, LDDM, and LDVL (Fig. 3C, Tables 1–2). These results show that the increased renewal induced by β-AR activation is accompanied by altered neuronal activation in a subset of brain regions, particularly in the prefrontal cortex and thalamus.

### Interregional connectivity patterns arise during renewal and are reshaped by β-AR activation

**Fig. 4:**
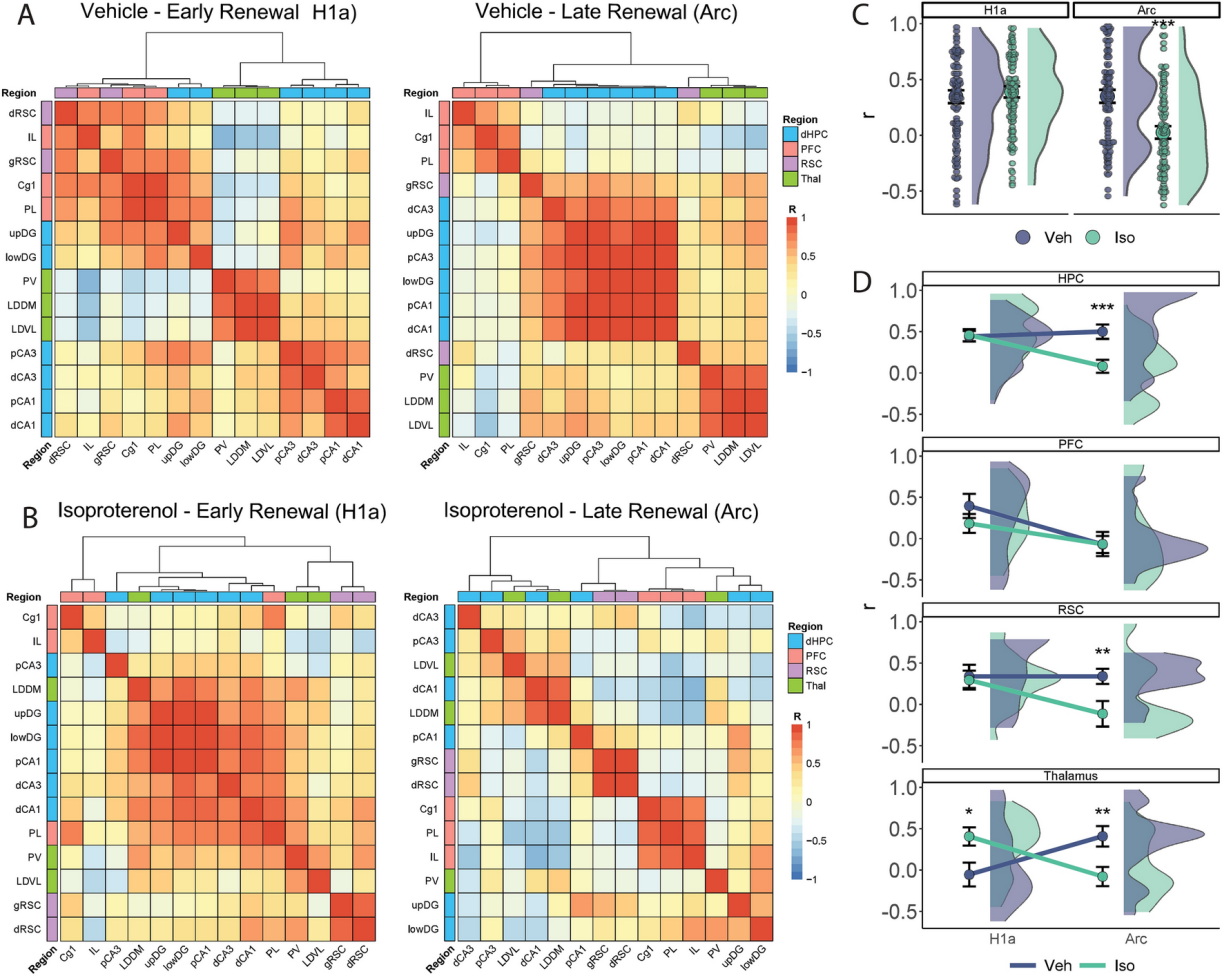
Patterns of interregional connectivity emerge during renewal testing and are changed by β-AR activation. Matrices showing the Pearson correlation coefficient values for each pair of structures and dendrogram showing subsequent hierarchical clustering for the **A**) Vehicle group (Veh) and **B**) Isoproterenol group (Iso). **C**) Overall group comparison of r values for Homer1a (H1a) and Arc. **D**) Between-group comparisons of r values derived from H1a and Arc expression across brain regions. Plots show mean ± s.e.m. Asterisks indicate statistical significance at *p < 0.05, ** p < 0.01, *** p < 0.001.

When the activation of different brain areas varies together, it can be assumed that they are functionally linked. To scrutinise the abovementioned brain-wide interactions during renewal further, interregional Pearson correlations were computed between all pairs of structures within each group and for each IEG (Supplemental Fig. 2). Subsequently, hierarchical clustering with Ward’s method^63^ was conducted to visualize brain areas that were close together and their relationships (Fig. 4).

In the vehicle group (Fig. 4A), during the early phase of renewal testing (reflected by Homer1a expression), we observed a large and varied cluster comprising the DG, RSC, and PFC, and separate thalamic and CA3-CA1 clusters. In contrast, during the late phase of testing (reflected by Arc expression), when extinction of the renewal response was evident, the hippocampus formed a larger intrastructural cluster that was distant from a thalamic and a PFC cluster, with the RSC divided between them.

In the β-AR agonist-treated group (Fig. 4B), during the initial phase of renewal (Homer1a expression), we observed a large cluster comprising the hippocampal subfields, LDDM, and PL, while other regions were relatively distant. However, during the later phase of renewal testing whereby under these conditions, renewal was sustained (Arc expression), a few pairs of structures showed large positive correlation coefficients, and clear clustering was observed only in the PFC and RSC. These findings suggest that the hierarchical relationships between brain regions changed throughout the renewal session and that these dynamics were affected by β-AR activation.

To assess differences in coordinated brain activation between groups and across time points during the renewal session, we first directly compared the respective r values in each condition (Fig. 4C). When looking at overall r values (across all regions), there was no difference between groups regarding Homer1a (p = 0.17). By contrast, for Arc, the r values were significantly decreased in the isoproterenol (β-AR agonist) group (U = 2,2648, p < 0.0001; Fig. 4.C). When specific anatomic regions in the early phase of renewal testing were assessed (reflected by Homer1a expression), we found that in the isoproterenol group the r values were significantly higher in the thalamus compared to the vehicle group (Mann–Whitney U test: U = 504, p = 0.01; Fig. 4.D). Regarding the late phase of renewal testing (Arc expression), the r values were lower in the isoproterenol group in the hippocampus (U = 4,643, p < 0.0001), RSC (U = 491, p = 0.005), and thalamus (U = 1,048, p = 0.004; Fig. 4.D).

These results suggest that β-AR activation initially leads to increased connectivity of dorsal thalamic nuclei, followed by a decrease in interregional coordination by the end of renewal testing, a time point where isoproterenol-treated animals displayed the highest recovery of extinguished responses (Fig. 1B).

### Functional networks supporting renewal are reshaped by β-AR activation

**Fig. 5:**
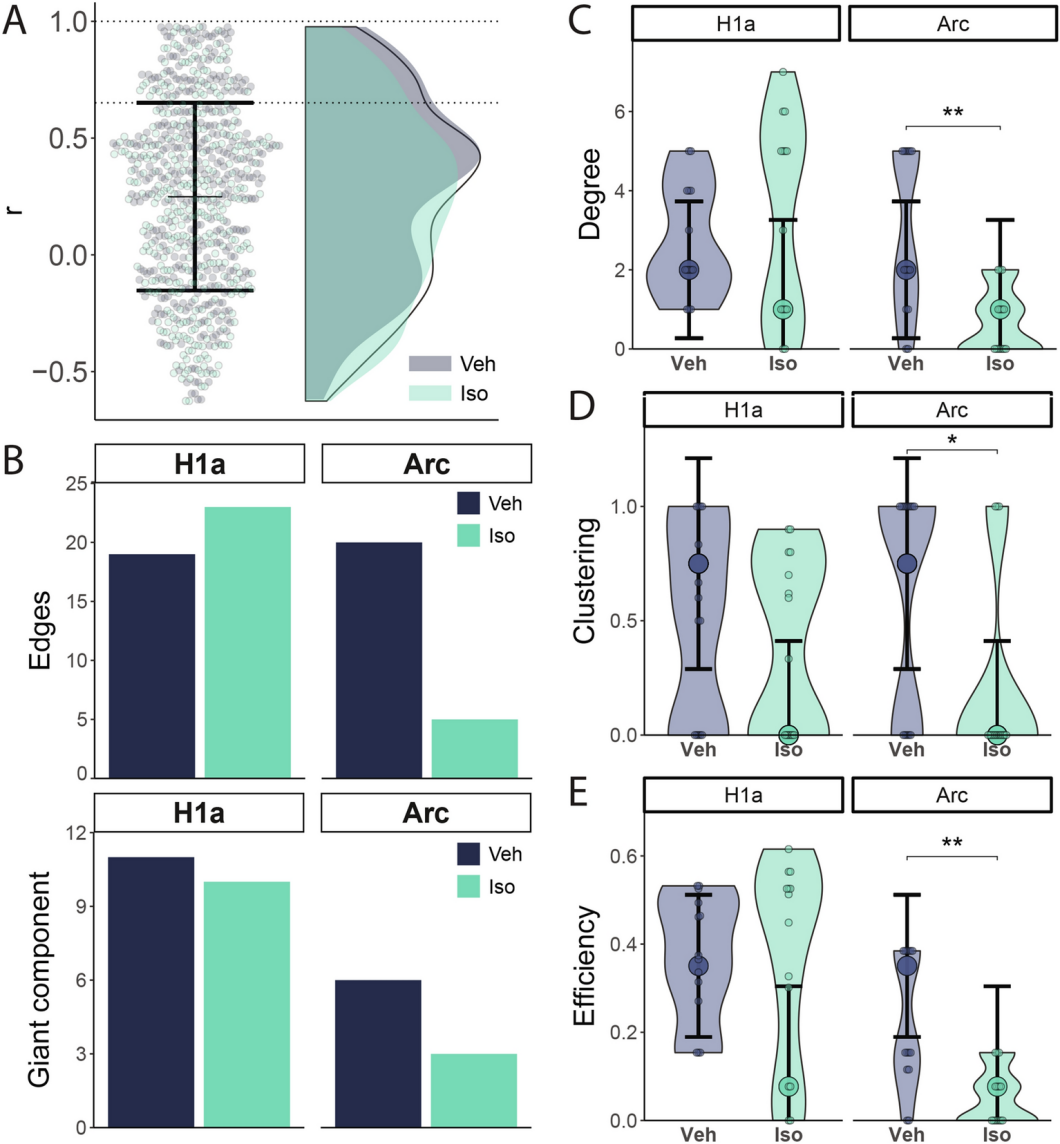
Centrality measures of renewal networks are impacted by β-AR activation. Functional network generation and centrality measures are shown in **A**) Distribution of r values and threshold based on the mean plus standard deviation. **B**) Top: Remaining edges following thresholding. Bottom: the size of the network’s Giant component following thresholding. **C**) Comparison of degree values between groups. **D**) Comparison of clustering coefficient values between groups. **E**) Comparison of nodal efficiency values between groups. Plots show mean ± s.e.m. Asterisks indicate statistical significance at *p < 0.05, ** p < 0.01, *** p < 0.001. H1a: Homer1a, Iso: Isoproterenol-treated group, Veh: vehicle-treated group.

To further investigate the differences in interregional connectivity, we used graph theory to generate functional networks. Accordingly, the data were first ‘thresholded’, so that only the strongest connections were retained. The cut point was determined as the mean plus the standard deviation of all r values (Fig. 5A). From the initial 91 connections in each group for each IEG, a similar fraction survived in both groups for the Homer1a network, but in the Arc network a smaller fraction was retained in the β-AR group (Fig. 5B, top). In addition, the giant component (size of the larger connected cluster) was similar between groups for the Homer1a network, but in the Arc network this metric was also decreased in the β-AR group, indicating the occurrence of increased modularity (Fig. 5B, bottom).

**Fig. 6:**
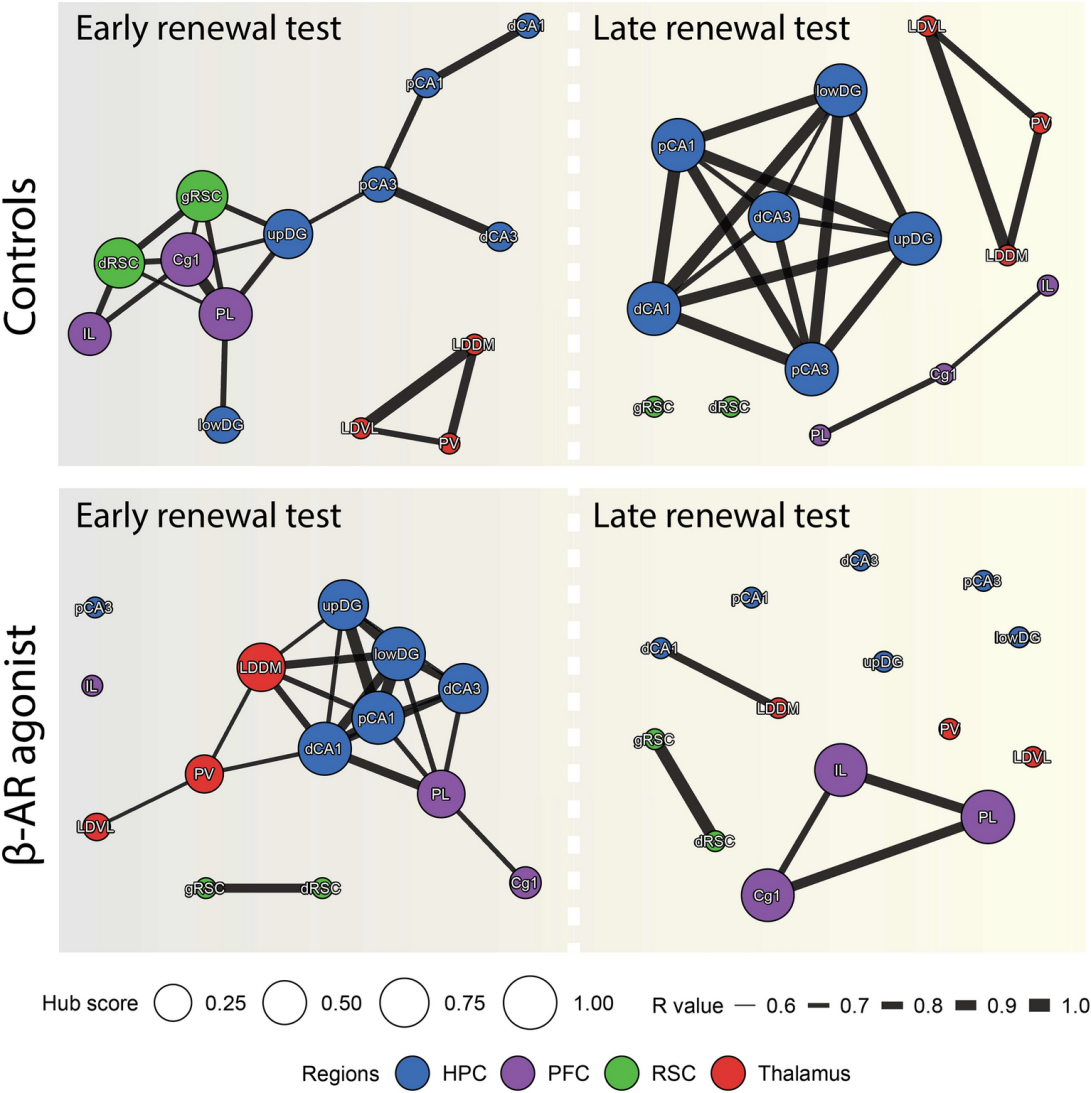
Functional networks supporting renewal are reshaped by β-AR activation. The schemes summarise the functional networks determined for each group of animals during early and late renewal testing. The width of the edges is scaled to the corresponding weight (r values), nodes are colored according to their respective brain regions and node sizes correspond to their relative hub score (eigenvector centrality).

To quantify and compare network properties, three centrality measures were utilized: degree centrality, clustering coefficient, and nodal efficiency (Fig. 5 C-E). Afterwards, functional network models were generated to visualize topological patterns in each condition (Fig. 6).

Degree centrality calculates the number of connections that a node has, and indicates the relative importance of each node within the network. Comparison of degree values revealed that although there were no overall degree differences between groups in the early renewal (Homer1a) network (Mann–Whitney U test: U = 87.5, p = 0.6,), there was a significant decrease in the β-AR group in the late renewal testing (Arc) network (U = 159, p = 0.004; Fig. 5C).

The clustering coefficient measures how tightly connected nodes form clusters, indicating the level of cohesion or modularity within the network. It was significantly lower in the β-AR group in the late renewal testing (Arc) networks (U = 140, p = 0.26; Fig. 5D).

Nodal efficiency provides information about how efficiently information flows through the network and how much information from a node reaches other nodes. There were no differences in efficiency between groups for the early renewal (Homer1a) networks, but it was significantly reduced in the β-AR group for the late renewal testing (Arc) networks (U = 163, p = 0.002; Fig. 5E).

Topological analysis of the networks revealed striking differences in connectivity patterns and hub regions between the vehicle and isoproterenol (β-AR agonist)-treated groups (Fig. 6). During early renewal (Homer1a) in the vehicle group, a tight PFC-RSC cluster connected with the hippocampus via the dentate gyrus, while the thalamus formed a separate cluster. At the end of renewal testing (Arc) where response extinction occurred in the vehicle group, the hippocampus formed a densely interconnected cluster, while the thalamus and PFC formed separate isolated clusters and RSC subregions seem to be acting in low coordination with the broader network.

In contrast, the isoproterenol group displayed a network centered around the hippocampus during early renewal (Homer1a), with the thalamic nuclei and PL and Cg1 forming part of the cluster, and the remaining structures being isolated. Such shifts in connectivity might underlie the apparent attenuation of hippocampal activity observed during early renewal based on overall Homer1a levels, although this could not be specifically addressed in the present study. At the end of the renewal testing session when renewal peaked (and no response extinction occurred) in the isoproterenol group (Arc), the network displayed sparse functional connections, indicating a scenario where structures were competing rather than cooperating during information processing.

These findings reveal the functional network dynamics underlying memory renewal and suggest that initial renewal in controls is dominated by memory retrieval dynamics within the hippocampus, supported by the RSC, coupled with decision-making activity within the PFC. As the animal realises that a reward will not appear, and extinction of the renewal response takes place as a result, the hippocampus becomes the sole hub of the functional network, presumably because information updating is taking place.

When β-AR activation occurs, altered coordinated activity between specific brain regions becomes evident. Initially, β-AR activation, which is coupled with an enhanced renewal response, causes an increase in connectivity between hippocampus, thalamus and PFC (without IL): a network that may signify attention and retrieval enhancement, coupled with reward expectation. At the end of the session, when renewal performance peaked, connectivity within and between the HPC, RSC, and dorsal thalamic nuclei was at its lowest point and the PFC formed the dominant network.

## Discussion

In this study, we aimed to elucidate to what extent different brain networks support renewal of spatial appetitive behavior and the subsequent extinction of this behavior in the absence of an appetitive reward. We also explored the extent to which β-AR activation influences this response. In control animals, we observed strong hippocampal-PFC-RSC connectivity during initial renewal that shifted to a predominantly insular intraconnectivity of the hippocampus during extinction of the renewal response, as the animal realised that no reward was available (Fig. 6). Strikingly, β-AR activation resulted in stronger renewal responses that failed to extinguish. Unlike controls, β-AR activation enabled an initial enhanced functional coordination between the hippocampus with thalamic nuclei, at the expense of synchrony with the IL and RSC, followed by a state of decreased interregional coordination (Fig. 6). These findings provide novel insights into network information processing during renewal, highlighting the essential role of the hippocampus in this process. They also show how state-dependent activation of β-AR can promote renewal by recruiting the thalamus and fragmenting information processing relevant for EL.

In line with previous studies on context-dependent modulation of spatial appetitive EL ^14,51,64–66^, we confirmed that the return of the animals to the acquisition context (A) on day 5, after successful EL (on day 4) triggered renewed search behavior in the goal arm, followed by extinction of the choice of the previously rewarded T-maze arm. While previous studies have reported larger renewal effects^14^, the effects observed here were more modest. We attribute this discrepancy primarily to differences in the interval between extinction learning and renewal testing (same-day vs. next-day) and, more importantly, to the T-maze setup introduced in the current study. The larger, elevated maze with transparent walls, enriched contextual cues, and dim lighting made animals more inclined towards exploratory behavior and arm alternation, reducing goal-directed responses when the reward was omitted (Supplementary Fig. 1A). To address this, we normalized correct responses to the first EL block—a time point when the appetitive memory was still intact despite the absence of reward (Supplementary Fig. 1B). This analysis supported a context-induced renewal effect, as indicated by the reduced correct responses during EL in context “B” and recovery upon the return to context “A”. Notably, full recovery occurred during early renewal testing in vehicle-treated animals, but only occurred during late renewal testing in isoproterenol-treated animals.

Interestingly, the increase in performance later in the renewal session caused by β-AR activation deviates from the common expectation that performance would peak earlier^14^. In line with the view that high NA levels enhance the processing of high-priority information while suppressing the rest^58^, we hypothesize that this may indicate that β-AR activation biases decision-making toward goals, while decreasing the detection of new contingencies, and as a result, goal-directed behavior progressively increased despite the absence of rewards.

We assessed nuclear expression of Homer1a to detect network activity during initial renewal, and nuclear Arc expression to detect network activity in the later phase of renewal testing^23^. This is based on knowledge that peak nuclear Homer1a expsession occurs 35–40 min after a behavioral experience^67^, whereas peak nuclear Arc expression occurs 5–6 min after a specific experience^68^. While FISH does not directly measure protein levels, given that mRNA expression patterns may not always correspond to translated protein, it offers a robust method for detecting neuronal activity through mRNA detection of IEG. Importantly, since our control animals (home cage group) were not subjected to the behavioral protocol and only provide information about baseline IEG expression levels, we cannot determine whether the observed changes in IEG expression are unique to renewal or also occur during other phases, such as acquisition or EL. Graph theory-based analysis^69–71^ was used to assess coordinated activity between multiple brain regions, based on the peak nuclear IEG expression detected.

The connectivity patterns observed throughout renewal testing provided valuable insights into the neural mechanisms underlying flexible memory expression and adaptation to changes in contingencies during spatial navigation. During initial renewal in control animals, somatic expression of Homer1a highlighted strong interconnectivity between the HPC, PFC, and RSC. This triad of activity suggests that past memories were being weighed against current experience^72–75^. We propose that this reflects the engagement of the hippocampus in the retrieval of the learned response, supported by the PFC that predicts the outcome of the behavioral responses^76^, while the engagement of the RSC provides a real-time representation of ongoing sensory experience related to spatial cognition^24^. Interestingly, the thalamus showed high intra-connectivity during initial renewal in control animals, indicating a state of focused attention. Despite this, thalamic nuclei were less integrated with the main HPC-PFC-RSC hub, suggesting a peripheral role in direct memory processing and navigation. Rather, the thalamus may have indirectly supported renewal by means of heightened sensory processing of visual stimuli^77^ that may have, in turn,  promoted RSC information processing^78^.

As the animals realised that no reward was available, failure of fulfilment of reward prediction resulted in extinction of the renewal response. Here, Arc expression revealed a different connectivity pattern: the hippocampal subfields became highly interconnected, albeit isolated from the brain regions studied here. Concurrently, the thalamus maintained its self-connection, as did the PFC, particularly centered in the cingulate cortex. This structure plays a key role in the accessing of remote memory^79,80^. This state therefore suggests that the animal engages in a reassessment and updating of internal models, based on a comparison of the retrieved remote memory (i.e. of the previously rewarded experience in ‘context A’) with the new contingency of unrewarded experiences (re-exposure to ‘context A’ after EL in ‘context B’). Extinction of the unrewarded renewal response might have also necessitated updating and reconsolidation of the memory of the context^81,82^, offering a possible explanation as to why the hippocampus acted alone during this phase of renewal testing, as indicated by the strong and isolated intraconnectivity of hippocampal subfield during this process.

In this study we identified a critical hippocampal-cortical-thalamic network that orchestrates the renewal of extinguished behavior. It is unsurprising that the hippocampus was involved in the renewal response, however. It is crucial for spatial navigation and rapid encoding and storage of information. The hippocampus also has the ability to discriminate and integrate across experiences, enabling it to form distinct, non-overlapping memories, as well as to promote generalization^83^. This capability makes the hippocampus vital in memory tasks that involve spatial or contextual components^84^, such as the rodent Water Maze^85^, T-Maze^86^, and contextual fear conditioning^87^. Given its capacity to form memory traces rich in contextual and spatial information, and to both integrate and segregate related stimuli, the hippocampus is also essential for EL and memory renewal^1,88^. Here, a novel finding of this study was that the initial renewal recruited activity in *all* dorsal hippocampus subfields. This suggests that initial renewal involves pattern separation^89,90^, pattern completion^91^, as well as the discrimination of spatial and non-spatial components of features of the T-maze^23,92–94^. The suprapyramidal blade of the dentate gyrus filled a central place in the network, connecting other hippocampal subfields with the cortex. This suggests that pattern separation, based on the discrimination of spatial cues^90,92^ was the primary driver of hippocampal information processing during initial renewal. Interestingly, the hippocampus became more active and more isolated during the extinction of the renewed response. This may reflect an engagement of the hippocampus in *de novo* information encoding, or in the substantial updating of the previously learned experience^83,95^.

Behavioral flexibility is essential for efficient navigation and interpretation of dynamic environments that contain shifting contingencies, as it allows animals to adjust strategies and update their learned behaviors^96^. This is supported by the RSC which plays an important complementary role in spatial cognition and memory, particularly in integrating egocentric and allocentric information and in the creation of manifolds of spatial representations, based on changing ambient conditions^24^. In line with this, during initial renewal in controls, a network that included the hippocampus, PFC and RSC was observed. During initial renewal we also found similar increases in nuclear IEG expression across hippocampal subfields and RSC in control rats. However, clear differences were observed in connectivity, suggesting the RSC primarily interfaced with the PFC and that it was the granular RSC that communicated specifically with the suprapyramidal blade of the dentate gyrus.

The granular RSC receives dense projections from the hippocampus^97^ and is the main recipient of hippocampal spatial and contextual information^35^. Accordingly, it also plays an important complementary role in spatial cognition and memory, particularly in integrating egocentric and allocentric information and in the creation of manifolds of spatial representations, based on changing ambient conditions^24^. In line with this, during initial renewal in controls, the RSC was well connected with the hippocampus (mostly particularly with the suprapyramidal blade of the dentate gyrus) and PFC. We speculate that its activity during initial renewal is likely to reflect its role in support of the retrieval of remote memory^98^.

The PFC significantly contributes to the suppression of disadvantageous behaviors, and to sustained attention to relevant stimuli^29,99,100^. Specifically, fear conditioning studies focusing on the infralimbic cortex (IL) have implicated this structure in EL^101–103^, and it has been proposed to act as a ‘brake’ on some learned motivated behaviors^29^. However, studies investigating other paradigms suggest a more complex role for the IL that involves the guidance of behavior according to changes in contingencies^29,104^. The prelimbic cortex (PL) has been reported to have a contrasting role to the IL^105^, whereby it promotes learning-associated motivated behaviors^106–108^. Yet, like the IL, its role appears to be contingency-dependent^109–111^. In control animals, during initial renewal, both PL and IL activity were integrated into a network with the hippocampus. This may indicate support by PL of reward prediction and arm selection, with regard to the expected reward location, whereas IL was initially engaged in the suppression of extinction learning. This network effect disappears in controls during extinction of the renewal response, when their behavior reflects that they realise that no reward will be made available.

Intracerebral treatment with the β-AR agonist was implemented via the lateral cerebral ventricle, meaning that systemic activation of cortical and subcortical structures is likely to have occurred. Furthermore, β-AR expression occurs in all regions of the brain^112,113^. Activation of β-AR, prior to renewal testing, resulted in a stronger renewal response than in controls. This response also failed to extinguish. Network analysis revealed that β-AR activation during initial renewal elevated the coupling of dorsal thalamic nuclei with the hippocampus and decreased synchrony with the IL and RSC. This is consistent with β-AR activation increasing arousal and attention^114^, to the disadvantage of information processing in the cortex. In the later phase of renewal testing, when it became apparent that renewal was sustained, information processing in hippocampus, RSC and thalamus became insular, operating in parallel to one another, and strong network interconnectivity became evidence only within the PFC. These findings suggest that the β-adrenergic receptor system can reroute interregional brain communication, prolonging this renewal by shifting hippocampal interactions away from cortical structures towards thalamic nuclei. As renewal intensifies under β-adrenergic influence, the brain transitions to a fragmented network state, where parallel processing dominates over the coordinated, integrative processing typically essential for memory retrieval and updating.

The manifestation of a network between hippocampus, PL and thalamus, during initial renewal following β-AR activation, was a very striking finding. Specifically, the LDDM, LDVL and PV were integrated into this network. The medial and lateral subregions of the dorsal thalamus are reciprocally connected to the PFC and have been implicated with a range of cognitive functions such as attention, inhibitory control, and memory^115^. For instance, it was reported in an attentional control task with cue-switching^116^ that the mediodorsal thalamus can reduce PFC interference, promoting context-relevant representations while suppressing irrelevant ones. Implementation of a choice serial reaction time task^117^ that allows the concurrent study of learning, attention, and impulsivity, results in inhibition of projections from the PFC to laterodorsal thalamus, decreased premature responses and increased omissions^118^, suggesting that this interaction might drive response actions and encode a “go” signal. Accordingly, it was reported in the same task that inhibition of PFC neurons projecting to the lateral mediodorsal thalamus, during conduction of the same task, decreased post-error response speed^119^, suggesting that the lateral mediodorsal thalamus can promote premature responses after unrewarded experiences. Additionally, evidence suggests that the PV is a critical node for goal-directed behaviors, serving to integrate information from prior experiences with interoceptive and exteroceptive signals^34^. Here, we found that concomitant with promoting memory renewal, β-AR agonism leads to an overactivation of the PV/LDDM/LDVL and increased its connectivity with the HPC, which might have biased the system to consider salient representations, previously associated with ‘Context A’, as relevant and those from ‘Context B’ as irrelevant, therefore boosting ‘go’ signals for reward-seeking.

A striking consequence of β-AR activation was that, in contrast to controls, the proximal CA3 was excluded from the network during initial renewal. The proximal CA3 may be mainly involved in the processing of non-spatial information^93,120^, suggesting that β-AR activation focusses information processing toward more spatial features of ‘context A’ that in turn enhances renewal. It may also indicate a failure of effective pattern separation during initial renewal in the presence of β-AR activation^121^.

While the hippocampus displayed connectivity with the PFC during initial renewal, it was disconnected from the IL and the RSC and connected with the dorsal thalamus during this response. Specifically, in contrast with controls, β-AR activation consistently isolated the IL: an effect have may sustained previously advantageous goal-directed behaviors, despite no reinforcement, suggesting a disrupted ability to adapt to new contingencies and to manage inhibitory control^122–124^. Moreover, IL activity was decreased in the later renewal testing phase when renewal responses peaked in β-AR agonist-treated animals. Curiously, the cingulate cortex adopted a relatively subordinate role during initial renewal in the presence of β-AR activation, also in contrast to its role in controls. Its conduit to the network was via the PL. This suggests that the retrieval of remote memory occurred on the basis of PL-mediated reward predictions^125^. Furthermore, the RSC exhibited an isolated node of activity under these conditions, indicating that it was excluded from hippocampal retrieval of the remote spatial memory^98^. These findings suggest that β-AR activation shapes brain networks into a configuration that favors the retrieval of reward-associated, positively-valenced, memory traces and reduces flexible changes in behavioral responses.

β-AR activation not only enhanced initial renewal, but also sustained this response through renewal testing. Network activity was radically changed, both compared to initial renewal in the presence of the agonist, and compared to responses in controls that exhibited extinction of the renewal response. All hippocampal, RSC and thalamic subfields, engaged in insular activity in the absence of network connectivity. Only two exceptions were evident: 1. communication between the distal CA1 subfield with the LDDM, consistent with attention being paid to (visuo)spatial cues^35,36^ and a focused goal-directed navigation strategy^126^, and 2. pronounced intraconnectivity between the PL, IL and cingulate cortex. This latter effect suggests that sustained renewal in the absence of a reward was possibly supported by reinforcement of remote memory^79,80^, in conjunction with suppression of EL by activity in the IL and reinforcement of reward expectation by PL^127^. Overall, such a reduction in functional connectivity might reflect a network configuration of competition, rather than cooperation, between functional modules that support an ‘act now, think later’ state.

Our findings highlight how state-dependent activity of the locus coeruleus-noradrenaline system (LC-NA) system can potently influence whether renewal is efficacious or not. Others have shown that the LC-NA system can orchestrate brain network configurations^128–130^ with effects being apparent in multiple species including mice^131^, monkeys^128^, and humans^132^. Cognitive demands^133^ and NA neuromodulation alter attention networks for goal-oriented stimulus processing^134^. Moreover, chemogenetic activation of the LC-NA system in mice enhances connectivity in regions involved with salience^131^, a response that is also dependent on β-AR in humans^132^. Atomoxetine, an NA reuptake inhibitor, increases functional connectivity within the frontal default mode network (DMN), including the cingulate, infralimbic, and prelimbic cortices, an effect associated with decreased connectivity of the retrosplenial cortex and hippocampus with DMN-associated cortices^135^. Atomoxetine also reduces global functional connectivity, particularly between regions from different functional modules^136^. These findings align with our observations that β-AR activation led to a decrease in interregional coordinated activity and measurements of functional connectivity, reflecting increased modularity in information processing.

## Conclusions

Our study investigated the extent to which different brain networks contribute to renewal of a spatial appetitive learning task, and whether these networks change when the animal realises that no reward is available. In control animals, we observed that during initial renewal, hippocampal connectivity with RSC and PFC predominates. This is followed, during extinction of the renewal response, by an elevation of coordinated activity within all subfields of the hippocampus to the exclusion of interactions with other structures. This might reflect a shift from memory retrieval on the backdrop of reward prediction, to memory updating driven by the new knowledge that the context no longer contains a reward. The activation of β-AR changed these outcomes, and resulted in reinforced renewal as well as prevention of extinction of the renewal response. At the network level, effects were accompanied by an initial enhanced functional coordination between the hippocampus and thalamic nuclei that occured at the expense of hippocampal synchrony with the IL and RSC. As the renewal effect intensified in β-AR agonist-treated animals, functional connectivity sharply declined, revealing a shift in connectivity patterns, and a network state of parallel processing within, rather than joint information processing, across relevant brain areas. Overall, these findings indicate that dialog between the hippocampus and (sub)cortical structures is needed for memory retrieval and its balance is gated by β-AR. By contrast, memory updating and consolidation occur during periods of reduced transtructural dialog and insular nodal activity, whereby the brain networks that engage in this process reflect the memory content that should be updated or consolidated. Taken together, our findings provide a new framework for understanding how brain regions communicate and reconfigure to support complex cognitive processes such as learning, memory, and decision-making, what could have significant implications for understanding and treating conditions where maladaptive memory renewal plays a role, such as in anxiety disorders and addiction.

## Methods

The study was carried out in accordance with the European Communities Council Directive of September 22nd, 2010 (2010/63/EU) for care of laboratory animals. All experiments were conducted according to the guidelines of the German Animal Protection Law and are reported in accordance with ARRIVE guidelines. The study was approved in advance by the North Rhine-Westphalia (NRW) State Authority (Landesamt für Arbeitsschutz, Naturschutz, Umweltschutz und Verbraucherschutz, NRW). All efforts were made to minimize the number of animals used.

### Subjects

Male Long-Evans rats (280–310 g, Charles River, Deutschland) were housed in individual cages in a temperature and humidity-monitored vivarium (21–23 °C, 50 ± 2% humidity) with *ad libitum* access to water and maintained on a 12 h light/dark cycle. Animals were weighed before commencing the study and maintained at 85% of their initial body weight. All experiments were conducted during the light phase. Each rat was handled for at least 5 days before the behavioral procedures.

### Surgery

Animals were anesthetized with sodium pentobarbital (52 mg/kg) injected intraperitoneally. Analgesic treatment was administered subcutaneously before and after surgery (Metacam, 0.2 mg/kg, Boehringer Ingelheim Vetmedica GmbH, Ingelheim am Rhein, Germany). Guide cannulae were bilaterally implanted in the lateral cerebral ventricles (icv; AP – 0.5 mm, ML ± 1.6 mm, DV 4.5 mm) as described previously^137^. The cannulae were kept in place by dental cement (Paladur, Haraeus Kulzer GmbH, Dormagen, Germany) forming a head stage that was tightly fixed to the skull with three stainless-steel screws. The rats were monitored daily during a 2-week recovery period, and subjected to regular health checks by a veterinarian, before beginning the behavioral procedures.

### Drugs and drug delivery

The β-AR agonist, isoproterenol hydrochloride (Tocris Bioscience, Bristol, UK), in a dose of a 2 mg/ml was dissolved in isotonic sterile saline 1 h prior to its use. It was infused bilaterally into the intracerebral ventricles (icv) 30 min before a renewal session, using internal cannulae connected to Hamilton syringes via plastic tubes, as described previously^137^. A total volume of 5 μl per hemisphere was infused at a rate of 1 μl/min. Injectors were left in place for an additional minute before removal, to ensure proper drug diffusion.

### Behavioral apparatus

The experiment took place in an opaque tent made of fiberglass fabric (3 × 3 m) with black walls and ceiling, and controlled LED illumination. Training and testing were conducted in an elevated T-maze (95 cm) with transparent acrylic walls (30 cm)^138^. The T-maze consisted of a starting box (22 × 20 cm) with a sliding door, a main corridor (70 × 20 cm), and two side arms (60 × 20 cm) that ended in goal-zone areas (22 × 22 cm) (Supplementary Fig. 1A). To ensure the reward pellets were not visible from a distance, the goal zones were equipped with small floor indentations into which the rewards were placed. The experiment used two different contexts: context "A" for the acquisition and renewal trials, and context "B" for the EL trials. The contexts differed in terms of the floor visual pattern, distal cues positioned outside the maze, a faint odor placed at the end of the goal zones, and the tent’s illumination^66^. The animals’ behavior was monitored using a video camera (Basler GemICam, Basler AG, Ahrensburg, Germany) and Ethovision XT software (v 13, Noldus, Wageningen, The Netherlands) for offline analysis. During habituation to the T-maze and acquisition trials in the "A" context, a food reward was present (see details below). No reward was present in the "B", or second exposure to the "A" context.

### Behavioral procedures

The task consisted of four phases: habituation, acquisition, EL, and renewal. As an appetitive reward, 0.5 cm^2^ sized chocolate pellets (Dr Oetker, Bielefeld, Germany) were used.

### Habituation

During habituation, the maze had a smooth plastic floor-covering that was distinct from the floors used in Context A and B, and no specific odor or spatial cues were available. On the first day of habituation, rats were placed in the starting box, the door between the box and the maze was opened, and they were allowed to freely explore the maze for 5 min with food rewards scattered in both side arms and goal-zones to encourage exploration. On the second day of habituation, rats completed two sessions of two trials each, with a 5-min break between sessions. The food reward was placed only in a small indentation on the floor in each goal zone. Rats explored the maze until they found the reward, or until 2 min had elapsed, after which time they were guided back to the starting position.

### Acquisition

The animals underwent three days of acquisition trials, each of which consisted of four blocks of five trials of maximally 2 min duration. Each trial was separated by an inter-trial interval of 15 s, while each block was separated by a five-minute pause.

One goal-zone area (e.g. right turn into one of the T-maze arms) was rewarded and visits to this location were scored as “correct choices”, while visits to the opposite arm (error-zone) were scored as “incorrect choices” (Supplementary Fig. 1B). The goal and error zones were predetermined for each rat and remained consistent for all trials. If the animal entered the error-zone, the exit to the main corridor was blocked, and the animal was contained in the non-rewarded arm for 40 s before being allowed to exit and return to the starting position. If a rat failed to leave the starting box for 40 s during a trial, the entrance door of the maze was closed, and the trial was terminated. If a rat returned to the starting box before reaching the end of either T-maze arm, the entrance door of the maze was closed, and the trial was terminated. If the animal did not reach the end of either T-maze arm within 2 min, it was guided back to the starting box, and the trial was terminated. All of these instances were scored as “incorrect choices”.

The probability of receiving a reward decreased during the acquisition phase, starting at 100% on the first day, 80% on the second day, and 60% in the first 10 trials of the third day, with a final probability of 40% in the last 10 trials of the third day. This approach was employed to avoid that the animals acquired a procedural learning strategy to learn the location of the reward^139^ and to increase perseverance in the spatial task^51,66^. Animals that did not achieve at least an 80% success rate (i.e. 80% correct arm choices) in the final trial block of day three were excluded from the study.

### Extinction learning phase

On the day immediately after the conclusion of the acquisition trials (i.e. on Day 4), the animals participated in EL trials in the same T-maze that contained modified contextual cues (Context “B”). The EL session consisted of 4 blocks of 5 trials, and the same criteria for scoring correct and incorrect choices used in the acquisition session were applied. No reward was available for any trial during the EL trials.

### Renewal testing

On Day 5, following the completion of the EL trials, the animals were divided into two groups and received icv infusions of either the β-AR agonist, isoproterenol, or the same volume of vehicle solution. Animals were distributed to either the isoproterenol, or vehicle, groups with the aim of ensuring that the percentage of correct choices during the acquisition and EL phases was similar between the two groups. Thirty minutes after the infusions, the rats were exposed to renewal trials with the same contextual cues as those used during the acquisition phase (Context “A”). The renewal session consisted of four blocks of five trials, and the same criteria for scoring correct and incorrect choices were applied as before. No rewards were available for any trial during the renewal phase.

Inter-trial and inter-block breaks were managed to ensure that the total time of the session was 40 min. This was done to ensure that somatic Homer1a could be used as a biomarker of initial renewal (somatic expression peak 35–40 min after behavior begins) and Arc could be used as a biomarker of processes occurring in the late phase of renewal testing (somatic expression peak 5–6 min after behavior begins)^23,67,68^. Animal behavior was scored by a researcher who was blind to the identity of the experimental groups.

### Fluorescence *in situ* hybrization

Forty minutes after starting renewal testing, brains were rapidly removed, flash-frozen in -40 °C isopentane, and stored at -80 °C^23^. Three animal groups were tested: **1**. Vehicle-treated animals, **2**. Isoproterenol-treated animals and **3**. Home cage controls. The home cage controls remained in the vivarium without disturbance for the duration of the experiment and had brain tissue collected using the same procedures and at the same time of day as the test animals (groups 1 and 2). Home cage controls served as a reference for basal IEG expression under conditions where no spatial behavior was instigated.

Coronal sections (20 μm) of the frozen brains were cut at -20 °C using a cryostat (CM 3050S, Leica, Wetzlar, Germany) and mounted on gelatinized slides. To facilitate proper localization of regions of interest, every 12th coronal section underwent Nissl staining. Somata for FISH analysis were stained with 4′,6-diamidino-2-phenylindole (DAPI)^23^. Once the regions of interest were identified, brain sections were treated with a previously established double fluorescence *in situ* hybridization protocol to reveal nuclear Homer 1a and Arc expression, as described previously^14,23^.

Images were acquired with an Axioscan.Z1 scanner (Carl Zeiss GmbH, Germany) and quantification was conducted through a semi-automated pipeline using QuPath (v 0.4.3)^140^. Initially, 300 × 300 µm annotations at the center of the regions of interest were created. DAPI-stained nuclei were detected via machine learning using StarDist2D^141^ and the pre-trained model ‘dsb2018_heavy_augment’ (github.com/stardist). Positive Homer1a and Arc puncta were detected using a pixel classifier with a Laplacian of the Gaussian prefilter, and thresholds were manually defined for each image and channel (i.e. Homer1a and Arc). A customized QuPath script classified the DAPI-stained nuclei as negative, Homer1a positive, Arc positive, or double-labeled. IEG expression was assessed by a researcher who was blind to the identity of the experimental groups.

### Statistics

We employed either a two-way analysis of variance (ANOVA) with repeated measures, or a one-way ANOVA to analyze the behavioral responses, and Homer1a and Arc expression, respectively. In cases where significant interactions were observed, Tukey’s post hoc tests were conducted to identify the specific differences.

### Network analysis

Aiming to gain a deeper insight into the interactions among brain regions, we leveraged graph theory to generate functional networks from brain activation revealed by nuclear Homer 1a and Arc expression. To assess the coordinated activity between brain regions, Pearson’s correlation coefficient was used. Mean r values between groups were compared with Mann–Whitney tests.

To generate functional networks, we retained only the strongest correlations as edges. Specifically, we derived the threshold by calculating the global mean plus one standard deviation of all pairwise correlation coefficients within each group x IEG combination. Any pairwise correlation above this threshold was considered an edge in the network, and the corresponding r value served as the edge weight. Nodes in these networks represent distinct brain areas, and edges represent the strongest correlations between them.

The R package igraph (igraph.org/r) and custom R code (github.com/johaubrich) were used to generate the networks and compute centrality measures. To evaluate network general structure and connectivity patterns, we calculated the network’s giant component, number of edges, degree centrality, nodal efficiency, and clustering coefficient. The giant component was defined as the largest connected subgraph of the network, encompassing the greatest number of nodes connected either directly or indirectly through edges. The size of the giant component was calculated as the number of nodes included in this largest connected subgraph. The number of edges within each network was calculated as the total count of connections (edges) that survived the thresholding process. Degree was calculated as the number of edges connected to each node. Efficiency was computed as the average inverse shortest path length from a given node to all other nodes in the network. The clustering was calculated using the local transitivity metric, which represents the ratio of triangles (closed triplets) connected to a node to the total number of triplets centered on that node. For nodes without neighbors, the transitivity was set to zero. We conducted Mann–Whitney tests to compare measures of degree, clustering coefficient, and efficiency. All statistical tests were two-tailed and the type-one error rate was set at 0.05. All statistical analyses and plots were generated using R version 4.2.2 (R Foundation for Statistical Computing, Vienna, Austria).

## Supplementary Information

Supplementary Information 1.

Supplementary Information 2.

## Data Availability

The code and data analyzed in this study are available at GitHub (github.com/johaubrich).
