# Supplementary material for: Cortico-subcortical networks that determine behavioral memory renewal are redefined by noradrenergic neuromodulation
Source: Sci Rep. 2025 Mar 20;15:9692. doi: 10.1038/s41598-025-93263-3 (PMC11926362; doi:10.1038/s41598-025-93263-3)
Supplement: Supplementary file 1 — Supplementary Information 1. [file 41598_2025_93263_MOESM1_ESM.pdf]

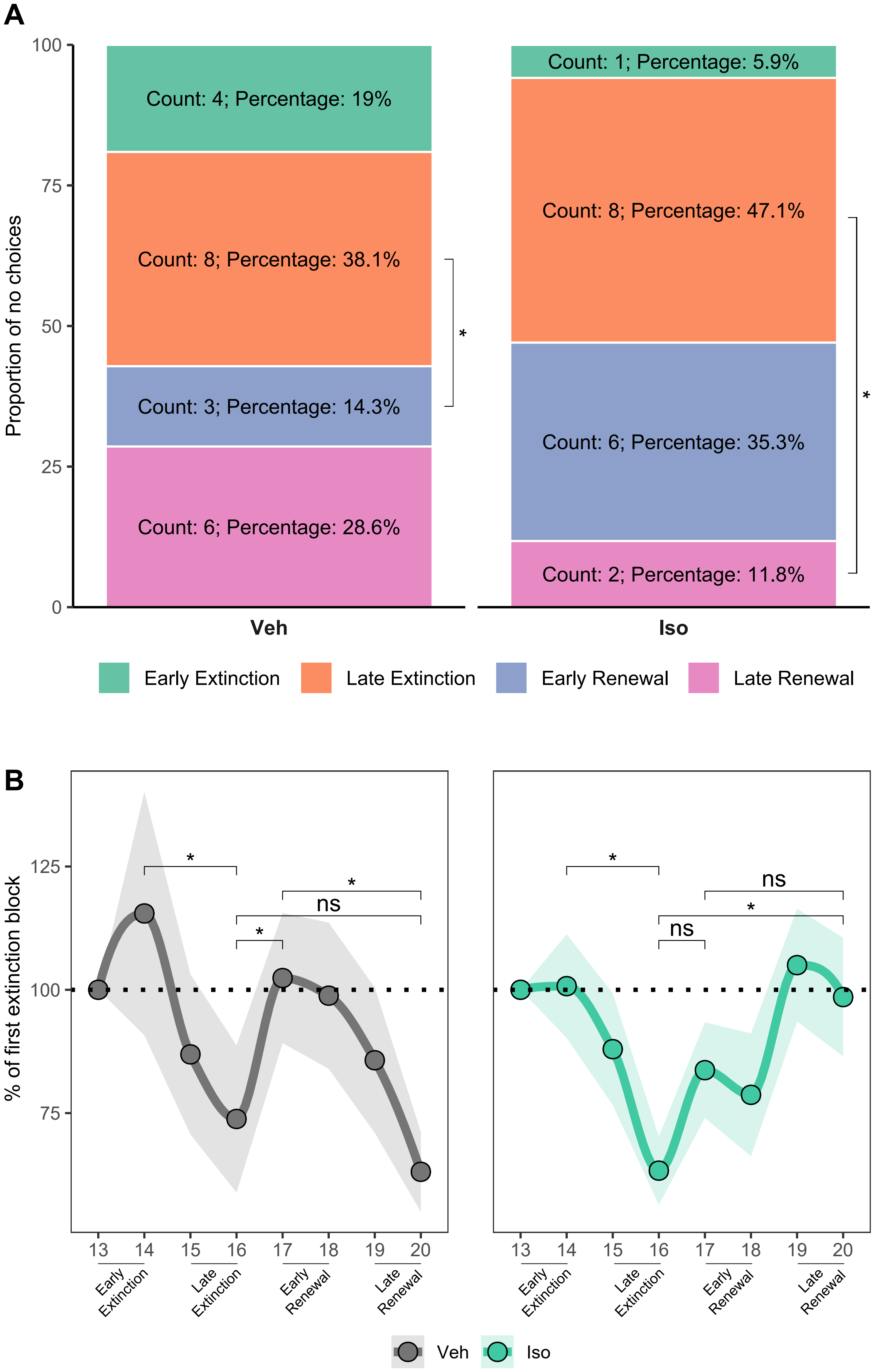

Supplementary Figure 1

A. Counts and percentages of trials with no arm entries (“no choices”) during unrewarded blocks (extinction and renewal sessions). The average individual proportion of “no choices” was compared between early (13-14) and late extinction blocks (15–16) and blocks in the early (17-18) and late renewal sessions (19–20) using pairwise Wilcoxon tests. Statistically significant differences ($p < 0.05$) between time points connected by brackets are indicated by asterisks.

B. Individual changes in correct responses across unrewarded blocks, normalized to each animal’s performance during the first extinction block. Dots represent group means, and ribbons indicate the standard error of the mean (SEM). Asterisks denote significant within-group differences ($p < 0.05$) between blocks connected by the brackets, based on Repeated-measures ANOVA followed by paired t-tests.
