# Supplementary material for: Cortico-subcortical networks that determine behavioral memory renewal are redefined by noradrenergic neuromodulation
Source: Sci Rep. 2025 Mar 20;15:9692. doi: 10.1038/s41598-025-93263-3 (PMC11926362; doi:10.1038/s41598-025-93263-3)
Supplement: Supplementary file 2 — Supplementary Information 2. [file 41598_2025_93263_MOESM2_ESM.pdf]

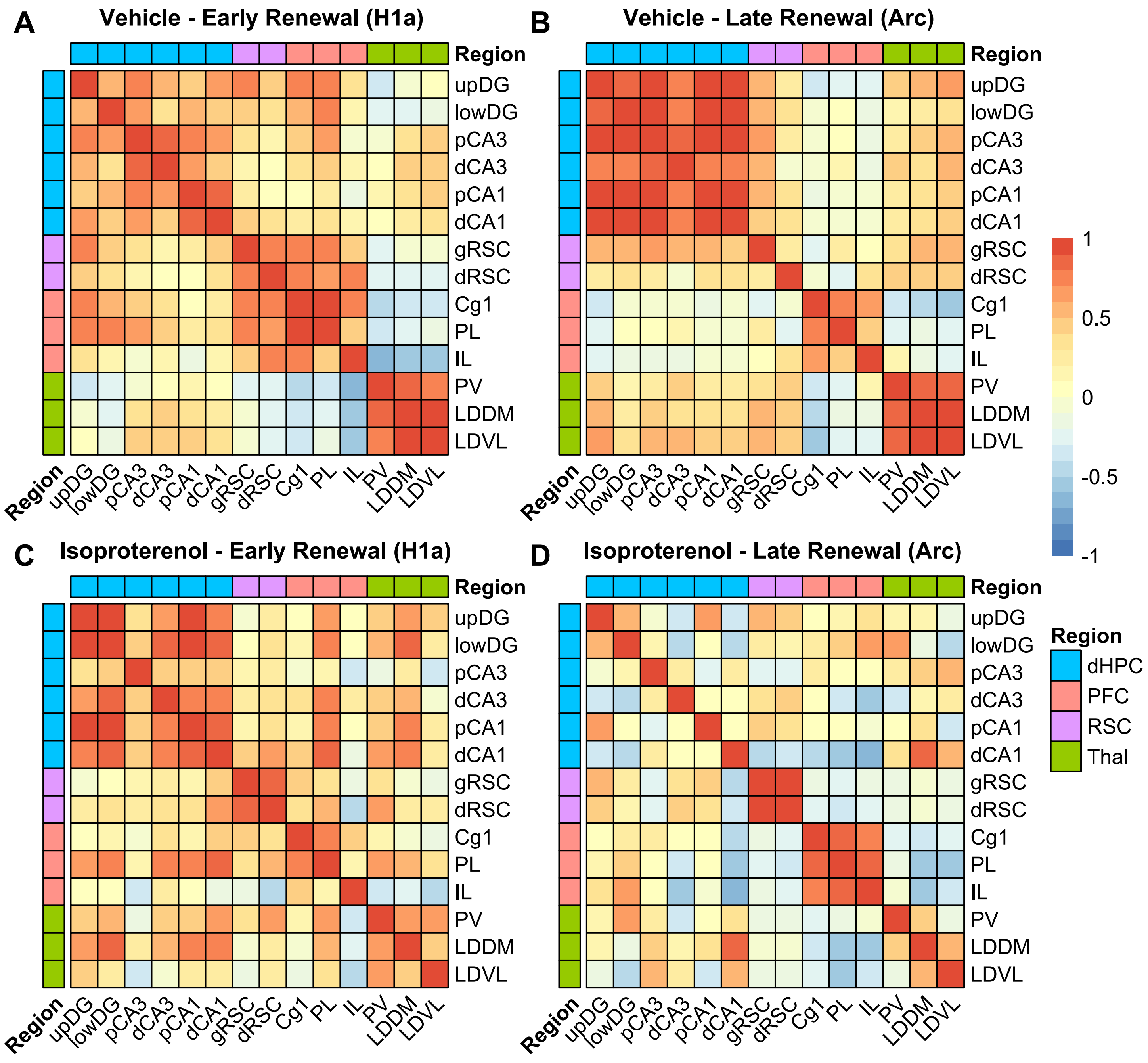


Supplementary Figure 2
Matrices showing the Pearson correlation coefficient values for each pair of structures as in Figure 4.A, but with structures in a fixed order (no hierarchical clustering applied).
